# Implications of insecticide resistance for malaria vector control with long-lasting insecticidal nets: trends in pyrethroid resistance during a WHO-coordinated multi-country prospective study

**DOI:** 10.1186/s13071-018-3101-4

**Published:** 2018-10-22

**Authors:** Implications of Insecticide Resistance Consortium, Jackie Cook, Sean Tomlinson, Immo Kleinschmidt, Martin James Donnelly, Martin Akogbeto, Alioun Adechoubou, Achile Massougbodji, Mariam Okê-Sopoh, Vincent Corbel, Sylvie Cornelie, Aurore Hounto, Josiane Etang, Herman Parfait Awono-Ambene, Jude Bigoga, Stanislas Elysée Mandeng, Boris Njeambosay, Raymond Tabue, Celestin Kouambeng, Etienne Fondjo, Kamaraju Raghavendra, Rajendra M. Bhatt, Mehul Kumar Chourasia, Dipak K. Swain, Sreehari Uragayala, Neena Valecha, Charles Mbogo, Nabie Bayoh, Teresa Kinyari, Kiambo Njagi, Lawrence Muthami, Luna Kamau, Evan Mathenge, Eric Ochomo, Hmooda Toto Kafy, Adam Ismail Bashir, Elfatih M. Malik, Khalid Elmardi, Jihad Eltaher Sulieman, Mujahid Abdin, Krishanthi Subramaniam, Brent Thomas, Philippa West, John Bradley, Tessa Bellamy Knox, Abraham Peter Mnzava, Jonathan Lines, Michael Macdonald, Zinga José Nkuni

**Affiliations:** MRC Tropical Epidemiology Group, Department of Infectious Disease Epidemiology, London School of Hygiene and Tropical Medicine, Keppel Street, London, WC1E 7HT UK

**Keywords:** Malaria, Vector control, Insecticide resistance, Trends, Bednets, Bioassay

## Abstract

**Background:**

Increasing pyrethroid resistance has been an undesirable correlate of the rapid increase in coverage of insecticide-treated nets (ITNs) since 2000. Whilst monitoring of resistance levels has increased markedly over this period, longitudinal monitoring is still lacking, meaning the temporal and spatial dynamics of phenotypic resistance in the context of increasing ITN coverage are unclear.

**Methods:**

As part of a large WHO-co-ordinated epidemiological study investigating the impact of resistance on malaria infection, longitudinal monitoring of phenotypic resistance to pyrethroids was undertaken in 290 clusters across Benin, Cameroon, India, Kenya and Sudan. Mortality in response to pyrethroids in the major anopheline vectors in each location was recorded during consecutive years using standard WHO test procedures. Trends in mosquito mortality were examined using generalised linear mixed-effect models.

**Results:**

Insecticide resistance (using the WHO definition of mortality < 90%) was detected in clusters in all countries across the study period. The highest mosquito mortality (lowest resistance frequency) was consistently reported from India, in an area where ITNs had only recently been introduced. Substantial temporal and spatial variation was evident in mortality measures in all countries. Overall, a trend of decreasing mosquito mortality (increasing resistance frequency) was recorded (Odds Ratio per year: 0.79 per year (95% CI: 0.79–0.81, *P* < 0.001). There was also evidence that higher net usage was associated with lower mosquito mortality in some countries.

**Discussion:**

Pyrethroid resistance increased over the study duration in four out of five countries. Insecticide-based vector control may be compromised as a result of ever higher resistance frequencies.

## Background

Vector control using indoor residual spraying (IRS) and insecticide-treated nets (ITNs) are core strategies for malaria control and elimination. The huge scale-up of these interventions in the last 20 years has been associated with major reductions in disease burden [1]. Between 2000 and 2015, it is estimated that over 1 billion ITNs were distributed in malaria endemic countries. The proportion of people in sub-Saharan Africa sleeping under a net increased from 30 to 54% between 2010 and 2016, whilst in 2016 an estimated 2.9% of the at-risk population was covered by IRS globally [1]. The increased coverage of vector control is estimated to have been a major contributor to the documented 62% decline in malaria mortality between 2000–2015 [2, 3]. However, between 2015 and 2016, data suggest that malaria mortality have remained the same in the WHO regions of Southeast Asia, the Western Pacific and Africa, and possibly increased in the Eastern Mediterranean and the Americas [1]. There are therefore justified concerns about the emergence and spread of insecticide resistance and the impact this may have on the continued effectiveness of insecticide-based interventions [1, 4].

Resistance has now been detected in malaria vectors to the four classes of public health insecticides used in malaria vector control (pyrethroids, organochlorines, organophosphates and carbamates) [5], and up to October 2016 had been reported in 71 malaria-endemic countries [6]. Until recently, pyrethroids have been the only class used for long-lasting insecticidal nets (LLINs) and accounted for a large proportion of the insecticide used for IRS. This heavy reliance on a single insecticide class prompted the World Health Organization (WHO) to issue a Global Plan for Insecticide Resistance Management (GPIRM) [5] which was subsequently expanded as part of the Global Vector Control Response [7]. The aim of these initiatives is to sustain the advances made in the fight against vector-borne disease through rational use of vector control tools, including insecticide deployment to slow the development of resistance. Country-level implementation of recommended activities and monitoring has been poor due to a combination of limited availability and costs of insecticides with new modes of action; human, financial and infrastructural capacity shortfalls; and insufficient data to determine epidemiological impact of insecticide resistance [8]. To address this latter point the WHO, with funding from the Bill and Melinda Gates Foundation, initiated a multi-country prospective study to assess the impact of insecticide resistance on the effectiveness of LLINs and IRS. The main objectives of the study were: (i) to determine the impact of insecticide resistance in malaria vectors on the protective effectiveness of LLINs and IRS, and hence on malaria disease burden; and (ii) to assess trends in the insecticide resistance status and underlying mechanisms in the main malaria vector species from the study areas in response to different interventions.

The study was conducted in five countries, Benin, Cameroon, India, Kenya and Sudan, with data collection conducted from 2010–2016. Details of the overall study design are given in Kleinschmidt et al. [9]. Overall epidemiological outcomes, presented in Kleinschmidt et al. [10], showed that nets provided protection against malaria irrespective of resistance frequency, indicating that populations in malaria endemic areas should continue to use LLINs to reduce their risk of infection. A number of country-specific analyses from this and other studies corroborate this finding [11–15]. In addition, several studies have published country-specific entomological data relating to the second objective [16–18], with ranges of resistance to pyrethroids reported. In this paper, we describe temporal and spatial trends in insecticide resistance of the main malaria vector species from across the five study countries.

## Methods

### Study design

The overall study design is described in detail in Kleinschmidt et al. [9]. The five countries included in the study were selected to represent areas of varying transmission intensity where resistance had previously been detected in malaria vectors (Table 1). In 279 study clusters (villages or groups of villages) across 16 areas in the five countries pyrethroid susceptibility in malaria vectors, and malaria infection and disease in children were measured simultaneously over several years. We aimed to assess whether higher levels of resistance are associated with loss of effectiveness of LLINs, and to characterise temporal and spatial trends in insecticide resistance. The numbers of clusters chosen per country are shown in Table 1 and were based on sample size calculations determined by the epidemiological outcomes [9]. Clusters were defined as villages or groups of hamlets with no less than 500 houses and were at least 2 km apart to avoid spill over in outcomes between clusters.

**Table 1:** Details of study sampling and sites including vector control coverage and insecticide resistance prevalence at baseline

	Study sampling sites				
	Benin	Cameroon	India	Kenya	Sudan
Malaria transmission intensity	High	High	Low	High	Low
Study locations	Districts of Ifangni, Sakété, Pobé and Kétou (Departement de Plateau)	Districts of Garoua, Pitoa and Mayo Oulo (North region)	Subdistrict of Keshkal (Kondagaon, Chhattisgarh)	Districts of Teso, Rachuonyo, Nyando and Bondo (western Kenya)	El Hoosh and Hag Abdalla (Gezira State); Galabat (Gedarif State; New Halfa (Kassala State)
Number of clusters sampled	32	38	80	61	79
Entomological sampling points (years)	2011–2015	2012–2015	2013–2016	2011–2015	2011–2014
Main malaria vectors	*Anopheles gambiae* (*s.s*.)^a^, *Anopheles coluzzii*^a^	*An*. *arabiensis*^a^, *An*. *gambiae* (*s.s*.)^a^, *An*. *funestus*	*An. culicfacies* ^a^	*An. gambiae* (*s.s.*)^a^*, An. arabiensis*^a^, *An*. *funestus*	*An. arabiensis* ^a^
Vector control interventions	High coverage of ITNs (primarily PermaNet 2.0) in all clusters	High coverage of ITNs (PermaNet 2.0) in all clusters	High coverage of ITNs (PermaNet 2.0) in all clusters	High coverage of ITNs (PermaNet 2.0 and Olyset Net) in all clusters. Rachuonyo and Nyando received IRS with deltamethrin and lambda-cyhalothrin in 2012, but no IRS was carried out subsequently	High coverage of ITNs (PermaNet 2.0) in all study clusters. In each study area half of clusters randomly allocated to receive additional IRS with bendiocarb
Baseline insecticide resistance information (cluster-specific range)	Kdr frequency by cluster ranged from 44 to 93% (2011) WHO Bioassay mortality to deltamethrin ranged between 20–100% (2011)	Kdr frequency by cluster ranged from 9 to 65% (2011) WHO Bioassay mortality to deltamethrin ranged between 43–100% (2012)	WHO Bioassay mortality to deltamethrin ranged between 86–100%	WHO Bioassay mortality to deltamethrin ranged between 1–100% (2011)	Kdr frequency by cluster ranged from 8.3 to 70.8% (2010); WHO Bioassay mortality to deltamethrin in sentinel clusters ranged between 47–100% (2011)

^a^Mortality results presented for these species in the analyses

### Vector control

LLIN mass distributions were carried out routinely in each site to provide universal coverage for each household (one net per two persons). Nets were distributed in Benin in 2011 (Olyset Net®, Sumitomo Chemical, Tokyo, Japan; 1 g/m^2^ permethrin) and 2014 (PermaNet® 2.0, Vestergaard, Lausanne, Switzerland; 55 mg/m^2^ deltamethrin), in Cameroon in 2011 and 2015 (PermaNet® 2.0), in India in 2014 (PermaNet® 2.0), in Kenya in 2010 and 2013 (PermaNet® 2.0), and in Sudan in 2011 and 2014 (PermaNet® 2.0). Net usage, defined as the proportion of respondents reporting as having slept under an LLIN the previous night, was determined through cross-sectional surveys which took place at least once in each country during the study period [10]. Cross-sectional household surveys, which consisted of sampling children from random households occurred in 2012 (Kenya, Sudan), 2013 (Cameroon, Sudan), 2014 (Sudan), 2015 (Benin, India) and 2016 (India) [10]. We used net usage as a proxy for the level of local mosquito exposure to pyrethroids. In Sudan half of the clusters were randomised to receive two rounds of IRS with bendiocarb (Ficam®80% WP, Bayer, Leverkusen, Germany; 200 mg active ingredient/m^2^). An exception was the Galabat region where clusters received IRS with deltamethrin (25 mg of a.i./m^2^; Chema Industries, Alexandria, Egypt) before changing to bendiocarb in subsequent years [15].

### Measuring resistance

Phenotypic susceptibility to the pyrethroid deltamethrin, in the main local vector(s), was measured annually in each cluster using WHO adult susceptibility tests and recorded as percent mortality [19]. In Benin, Cameroon, Kenya and Sudan larvae were collected from breeding sites within each cluster annually and reared to adulthood in insectaries. In India, where larval sites were difficult to locate, resting females were caught [19]. Adult female mosquitoes [of unknown age (India); 2–5 days-old (all other countries)] were exposed for 60 minutes to deltamethrin using WHO impregnated papers at standard concentrations (0.05% deltamethrin). Mosquitoes were kept at temperatures between 23 and 27 °C, with humidity, where measured, between 75–85%. Mortality was measured 24 h post-exposure. In all tests, observed mortality in control mosquitoes was less than 5% therefore Abbott’s correction was not applied.

### Statistical analysis

Mosquito mortality data were analysed at the level of the individual mosquito, with post-exposure status (dead/alive after 24 h) modelled as the response variable in logistic regression. Explanatory variables of interest were year, years since last LLIN distribution, and cluster- and year-specific LLIN use as measured in cross-sectional household surveys. Susceptibility test data were excluded from the analysis if fewer than 40 mosquitoes were tested. A mortality estimate was calculated per cluster for each time point, with data for each country analysed separately and in an all-country model. Association between cluster mortality estimates was assessed between years using binomial generalised linear models. Separate generalised mixed-effect models were used to assess trends in mortality over time, effect of time since LLIN mass distribution and effect of bednet use, with the cluster specified as the random effect to account for within cluster correlation of responses. Year was modelled as a linear term to investigate trends over time. Where appropriate, a regional identifier was included as a fixed effect to allow for spatial differences in resistance within countries. Where data were available, insectary temperature and humidity during resistance testing were included in country-level models (Cameroon, India, Sudan).

Cluster-level net usage, as a categorical variable (low, < 40%; medium, 40–80%; and high, > 80%), was explored as an explanatory variable in the years where these data were available from concurrent cross-sectional surveys. As bednet usage was only available for some years, a time variable was not included in these models. To investigate whether the impact of bednet distributions waned over time, models using time since bednet distribution (in years) as the key explanatory variable (as opposed to calendar time) were also examined.

Data from all 5 countries were combined to investigate whether there was evidence for an overall temporal trend in phenotypic resistance, with country added as a fixed effect. As the only data available from 2016 were from India, the all-country analysis was undertaken with and without India.

Results are presented in terms of changes in mortality of mosquitoes by year [Odds Ratios (OR) per year] or with increasing cluster-level category of net usage, with a reduction in mortality indicative of increasing resistance frequency.

## Results

### Estimates of mortality

More than 90,000 mosquitoes were tested in 911 separate tests across 5 countries and over 6 years. The median number of mosquitoes exposed per cluster per year was 100 [interquartile range (IQR) 84–104]. Median mortality across all tests was 81% (IQR: 63–94%). Insecticide resistance, classified according to the WHO criteria of < 90% mortality, was detected in all tested species, in all five countries and in 87% (*n* = 793) of tests performed. In only 7% of tests performed (*n* = 63, from 57 clusters) was 100% mortality observed. There were noticeable differences in the proportions of clusters defined as susceptible across countries. For example, in India, ≥ 98% mortality was observed in 28% (*n* = 66) of tests compared to only 1% of tests (*n* = 2) in Sudan. In Benin, Cameroon, Kenya and Sudan, > 50% mortality was recorded in at least 14% of tests recorded; no tests in India had less than 50% mortality.

### Temporal and spatial variation

**Fig. 1:**
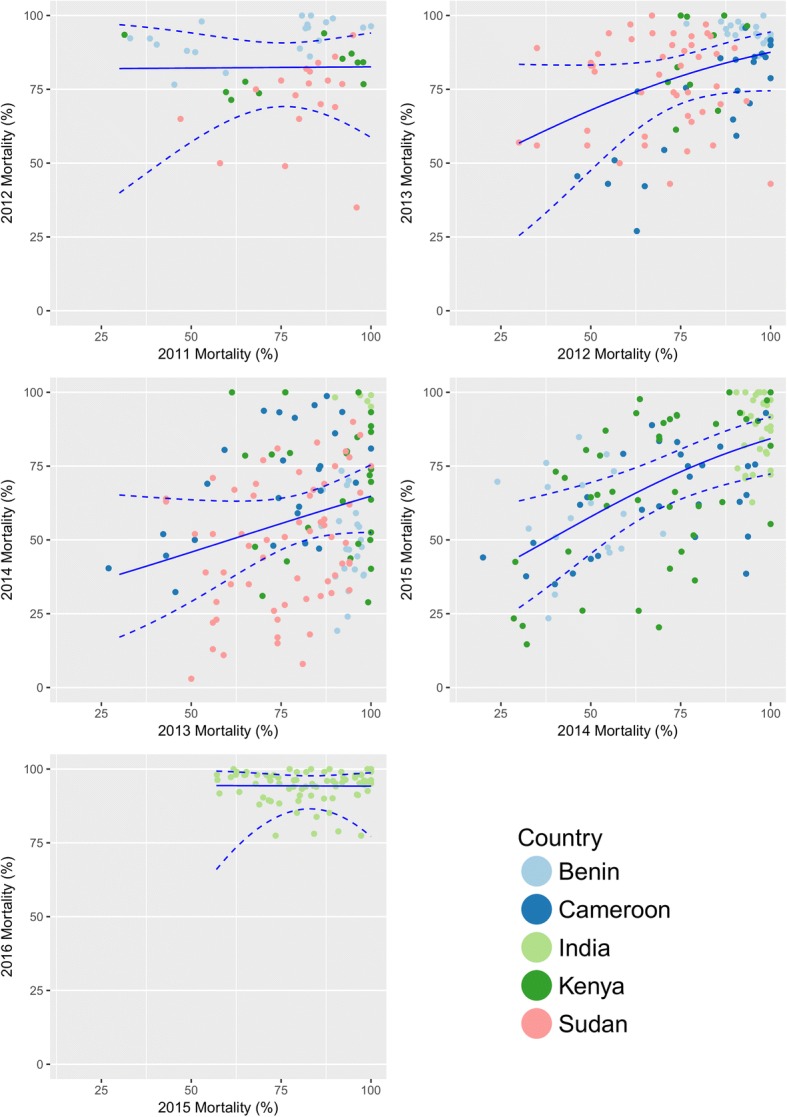
Association in cluster mortality between years. Scatter diagrams show results for clusters with mortality estimates in consecutive years for each year of the study. The predicted mortality result from binomial generalised linear models is overlaid on each graph with 95% confidence intervals

Cluster-specific mosquito mortality showed limited and inconsistent evidence of year-to-year correlation in all countries (Fig. 1). The strongest association was seen between data points from 2014 and 2015 (Kendall’s tau coefficient: 0.42, *P* < 0.001), although this pattern differed by country, with no correlation seen between those years in Benin or India (Sudan ceased data collection in 2014) (Kendall’s tau coefficient 0.07, *P* = 0.677, and 0.02, *P* = 0.886, respectively). The strongest correlation between years was seen in Cameroon, with Kendall’s tau coefficient > 0.3 for all year pairs (*P* < 0.02), whilst for the other countries correlation was only present in some pairwise comparisons.

### Trends in mortality over time

**Fig. 2:**
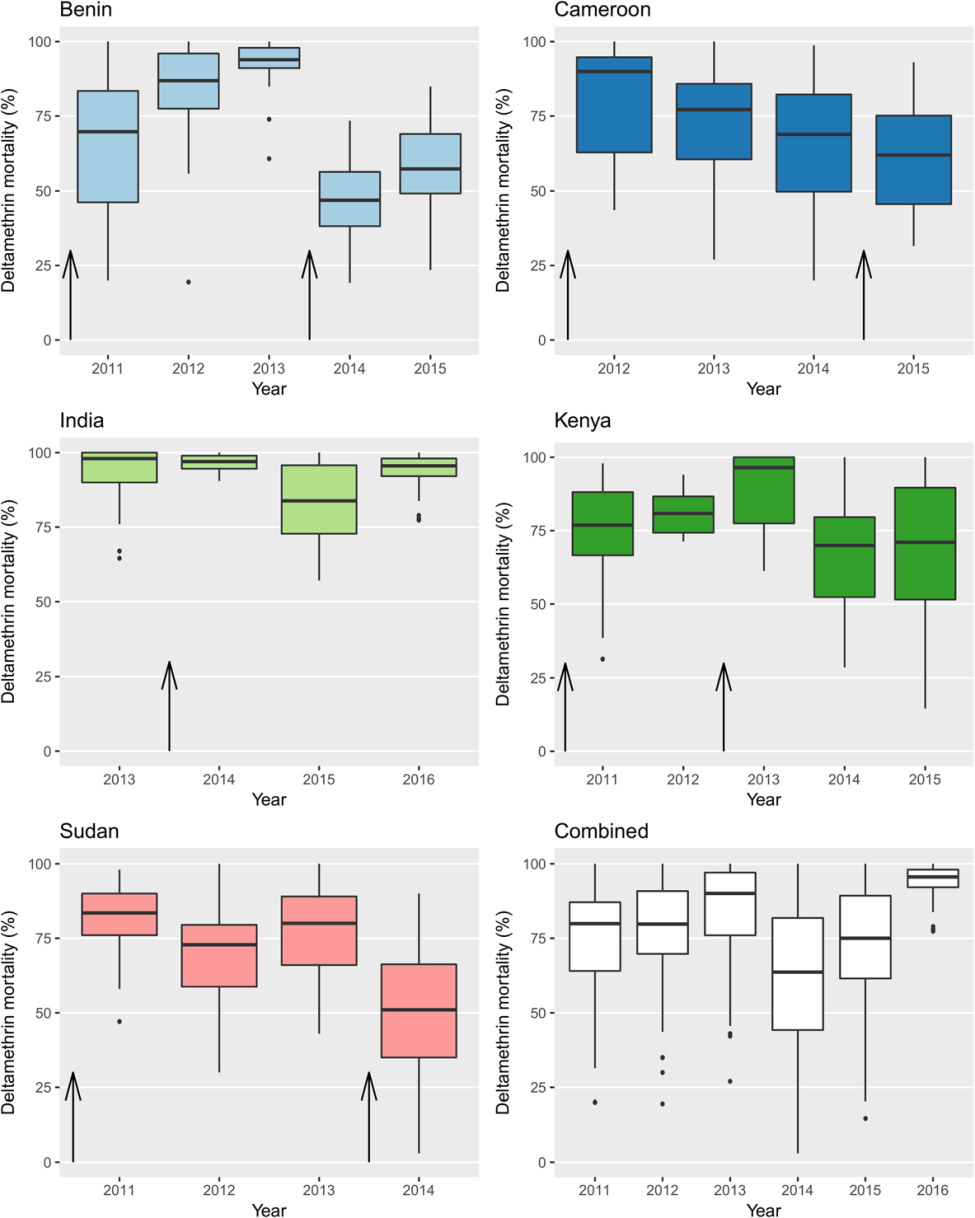
Box-and-whisker plots showing the range of cluster-level mortality by year and country. Arrows indicate the timing of bednet distributions within country

The trends in mortality over the study period differed by country (Table 2, Fig. 2). A decrease in mortality was detected in Benin, Cameroon, Kenya and Sudan. A slight increase in mortality was detected in India [aOR: 1.03 (95% CI: 0.98–1.1), *P* = 0.08]. The most substantial yearly decrease was detected in Sudan [aOR: 0.67 (95% CI: 0.64–0.70), *P* < 0.001]. With data from all countries combined, a 21% decrease per year in odds of mortality was detected [aOR 0.79 (95% CI: 0.79–0.81), *P* < 0.001]. This was not substantially altered with the exclusion of India [aOR 0.77 (95% CI: 0.76–0.79), *P* < 0.001].

**Table 2:** Impact of time on mosquito mortality. Results from generalised linear mixed-effect models examining the impact on mosquito mortality over time (year)

Country	Odds ratio for change in mortality per year (95% CI)	*P*-value
All five countries combined^a^	0.79 (0.79–0.81)	<0.001
Four countries combined (without India)^a^	0.77 (0.76–0.79)	<0.001
Benin^b^	0.74 (0.72–0.76)	<0.001
Cameroon^c^	0.74 (0.69–0.78)	<0.001
India^c^	1.03 (0.98–1.10)	0.08
Kenya^b^	0.88 (0.86–0.90)	<0.001
Sudan^c^	0.67 (0.64–0.70)	<0.001

^a^Adjusted for country

^b^Adjusted for district

^c^Adjusted for district, temperature and humidity

Results are presented in terms of change in odds of mortality of mosquitoes in WHO bioassays by year. Odds ratios are adjusted for locality and temperature and humidity where indicated. The data are shown for each country, as well as all countries combined (with country included as a covariate). Cluster was included as a random effect in all models

### Effect of bednet distributions and bednet use

Bednet distributions occurred in all sites during the study period. Associations between bednet usage and cluster specific mosquito mortality was investigated for each year that epidemiological cross-sectional data were available. Mean net use was above 65% in all countries, with Kenya reporting the highest value (94.2%). Benin, India and Kenya had no clusters with less than 40% net usage. Net usage appeared to have differential impact on mosquito mortality in each country with no association found in Benin and Kenya (*P* = 0.225 and *P* = 0.241, respectively); higher mortality found in areas with higher net usage in Cameroon (aOR 1.6 and 1.4 for net usage between 40–80% and above 80% respectively, compared to clusters with net use under 40%, *P* < 0.001) and strong negative associations found in India and Sudan (Table 3).

**Table 3:** Impact of cluster-level bednet usage on mosquito mortality. Results from generalised mixed-effect models examining the impact of cluster-level bednet usage on mosquito mortality

		All countries combined^a^	Benin^b^	Cameroon^c^	India^c^	Kenya^b^	Sudan^c^
	No. of clusters included (year)	59 (2012); 87 (2013); 143 (2014); 99 (2015); 80 (2016)	19 (2015)	22 (2013); 26 (2014)	80 (2015); 80 (2016)	13(2012); 41 (2014)	46 (2012); 65 (2013); 76 (2014)
	Mean net usage (range) (%)		74.9 (52.5–100)	67.8 (7.0–100)	89.9 (60.9–100)	94.2 (73.7–100)	78.6 (0–100)
Effect of cluster-level net usage on mosquito mortality, OR (95% CI)	< 40%	1 (reference)	–	1 (reference)	–	–	1 (reference)
	40–80%	1.03 (0.89–1.19)	1 (reference)	1.61 (1.21–2.14)	1 (reference)	1 (reference)	0.69 (0.58–0.83)
	> 80%	0.65 (0.57–0.74)	1.59 (0.75–3.37)	1.40 (1.08–1.82)	0.36 (0.29–0.44)	2.38 (0.56–10.1)	0.45 (0.38–0.53)
	*P*-value	<0.001	0.225	<0.001	<0.001	0.241	<0.001

^a^Adjusted for country

^a^Adjusted for district

^c^Adjusted for district, temperature and humidity

Results are presented in terms of change in mortality of mosquitoes for increasing bednet usage category (< 40%; between 40–80%; and above 80%). Bednet usage was calculated for years where cross-sectional survey data was available. Odds ratios are adjusted for locality and temperature and humidity where indicated. The results are shown for each country, as well as all countries combined (with country included as a covariate). Cluster was included as a random effect in all models

Time since bednet distribution was also investigated to establish whether changes associated with bednet distributions waned over time. Differential trends were evident with Benin, India and Sudan demonstrating an increase in odds of mortality (decreasing resistance frequency) for each year post-distribution (*P* < 0.001 for each) whereas mosquito mortality in Cameroon (aOR: 0.95, *P* = 0.016) and Kenya (aOR: 0.59; *P* < 0.001) decreased (increased resistance frequency) with each year post-LLIN distribution (Table 4).

**Table 4:** Impact of time since bednet distribution (years) on mosquito mortality. Results from generalised mixed-effect models examining the impact time since bednet distribution on mosquito mortality

Country	Odds ratio for change in mortality per year (95% CI)	*P*-value
All countries combined^a^	1.34 (1.31–1.37)	<0.001
Benin^b^	3.20 (3.02–3.39)	<0.001
Cameroon^c^	0.95 (0.90–0.99)	0.016
India^c^	1.62 (1.52–1.73)	<0.001
Kenya^b^	0.59 (0.56–0.62)	<0.001
Sudan^c^	1.60 (1.53–1.67)	<0.001

^a^Adjusted for country

^b^Adjusted for district

^c^Adjusted for district, temperature and humidity

Results are presented in terms of change in mortality of mosquitoes for each year since a mass bednet distribution took place in-country. Odds ratios are adjusted for locality and temperature and humidity where indicated. The results are shown for each country, as well as all countries combined (with country included as a covariate). Cluster was included as a random effect in all models

## Discussion

Insecticides have been a key component in the public health and agriculture toolbox for over a century, resulting in the inevitable emergence of resistance in mosquito vectors. This study brings together a very large collection of data from a range of transmission settings to investigate the trends in pyrethroid resistance. Whilst year to year variation was substantial, and poor inter-year correlation prevented cluster specific predictions of resistance, a decrease in mosquito mortality was detected in four out of the five countries over the 5-year period of the study suggesting that resistance to pyrethroids has been gradually increasing in these settings.

WHO encourages regular monitoring of resistance frequencies to all insecticides used in country. Consequently, the level of reporting has increased dramatically in recent years with over 30,000 data points now entered into global databases such as the WHO Malaria Threats Map [20] and IR-mapper (www.irmapper.com) [21]. The picture that emerges from these summary data [6, 22, 23], as with the present study, is that resistance to pyrethroids is increasing in frequency and geographic extent. However, these global databases often aggregate data with substantially differential sampling effort across years and regions [6] which may obscure the substantial stochasticity in mortality estimates.

It is assumed that the increase in resistance to pyrethroids over the past decade is due in part to the higher coverage of insecticide-based interventions, such as LLINs. However, studies have shown conflicting results with some reports of increasing resistance following bednet distributions [24–27], and other reports of no increases despite sustained insecticidal campaigns [28–30]. Although ascertaining the effect of bednet coverage was not a primary goal of this study, it was possible to investigate the impact of net use through cross-sectional surveys that were conducted concurrently to resistance measurements. Trends were not uniform across countries, perhaps in part reflecting the differing biology of the vector species. *Anopheles arabiensis* (a major vector in Kenya, Cameroon and Sudan study locations) and *An. culicifacies* (primary vector in India study locations) commonly show high rates of zoophily. Obtaining blood meals from sources other than humans means LLINs would potentially have less impact on selective pressure for resistance. However, overall, higher net usage was associated with increasing resistance in mosquitoes. This trend was most evident in Sudan where the widest range of net usage was reported whereas in other settings reported net usage was more uniform, thereby reducing the likelihood of detecting a trend.

We did not discern a consistent trend in mosquito mortality with increasing time post-net distribution. In Benin, India and Sudan, mortality increased every year post-distribution, suggesting that the initial increased coverage of nets may have been a short-term driver for resistance and that as the insecticide on the nets reduced over time, the selection pressure reduced, in turn reducing the proportion of resistant mosquitoes. However, in Cameroon and Kenya, the opposite effect was observed, with mortality decreasing with every year from the date of the distribution. Data from the *An*. *gambiae* 1000 genome project has revealed that there appear to be numerous instances of localised adaptation to insecticide pressure [31]. The difference we observed in response to LLIN distribution may reflect in part this innate difference of vector populations to respond to insecticide pressure and caution against making generalised predictions.

Moreover, whilst bednet distributions will have increased selection pressure in the study settings, it is also possible that the insecticide resistance could be linked to ongoing agricultural practices [32–34]. In several African countries, including northern Cameroon, the use of pyrethroids for cotton farming has been implicated as a catalyst for the increase in recorded resistance in *An*. *gambiae* populations [34–36]. Differences in the use of pyrethroids for agricultural purposes in the study settings could further impact the relationship between time of net distribution and insecticide resistance.

Previous studies have also shown resistance to be highly focal [16, 37–39], with large variations over small geographical distances. This is exemplified by the range of mortality measures within each country and highlights the need for multiple sentinel monitoring sites per country and reinforces that extrapolating resistance data from few, widely-dispersed sentinel sites to larger areas is untenable. Spatial heterogeneity in insecticide resistance poses challenges for integrated resistance management and suggests that locally tailored vector control and resistance management programmes are required.

There was considerable temporal heterogeneity with high between year variation at cluster level. This phenomenon has also been reported elsewhere [40–43]. There are several reasons why levels of resistance in a mosquito population may fluctuate over time, for instance, resistance can recede if proper resistance management practices are implemented or if resistance drivers reduce and resistance associated genetic variants are deleterious in the absence of selection pressure [5, 44]. It is possible that in our study settings varying exposure to pyrethroids resulted in fluctuating frequency of resistance in the mosquito population with evidence from some areas suggesting that bednet usage resulted in higher resistance frequencies.

As well as genuine fluctuations in the frequency of resistance, it is possible that the different susceptibility recorded is, in part, an artefact of the method of testing. Longitudinal monitoring is easily influenced by any changes in protocol for measuring mortality and the timings of the tests. Some studies have demonstrated fluctuations in mosquito mortality over a transmission season [45] and whilst all efforts were taken to ensure that tests occurred at the same time each year, differences between seasons may have had an impact. In addition, humidity and temperature are known to have an impact on mortality testing [46]; whilst these were controlled for where data were available, it is possible that differing conditions influenced mortality results.

There is mounting evidence that tests recording mosquito mortality after 24 h may not be the best way to record changes in population resistance, particularly when the level of resistance is high [47]. A number of alternative options are now available for monitoring the presence of resistance, including molecular assays, time/dose response assays and increasing the time post-exposure at which mortality is calculated, all of which are likely to be more sensitive to resistance trends [48–51], but these methods are also more resource intensive. Although as noted by Churcher et al. [4] the strong association between bioassay data and mortality measured in experimental hut trials still supports the use of bioassays as a quantitative test of the impact of resistance on LLIN efficacy. In this multi-country study, to ensure comparability between sites, the test was performed using one insecticide dose and one exposure time, using wild-caught mosquitoes reared in the laboratory. These settings may not reflect adequately the conditions wild mosquitoes experience, such as variations in temperature, food availability and pre-existing pesticide exposure [51]. In addition, the doses used in the resistance tests are not necessarily reflective of the doses mosquitoes would experience in the wild, which can be influenced by age or retreatment of ITN or regularity and coverage of IRS. The dose used for detecting resistance can have a particularly strong effect depending on the prevalence and penetrance of the resistant mechanisms present in the mosquito population. Recording mortality at 24 h may also miss some of the nuances involved with the evolution of resistance which may result in delayed mortality [51]. In addition, mosquito age has been shown to have a big impact on susceptibility, with older mosquitoes showing higher mortality rates compared to their younger counterparts [52]. If insecticides remain effective against mosquitoes old enough to transmit malaria, this may explain why some studies are observing minimal impact on epidemiological outcomes [10–12, 14].

## Conclusions

This study demonstrated increasing frequency of resistance to pyrethroids in malaria vectors from 4 out of 5 study countries. Although the increase does not appear linear, if the current trend continues, it is likely to result in a reduction of the effectiveness of pyrethroid-based interventions such as ITN and IRS. There was evidence in some countries of increased selection pressure for pyrethroid resistance in clusters where net use was higher. There are a number of strategies presented within GPIRM to mitigate the increase of insecticide resistance in malaria vectors such as rotations, combinations, mosaics and mixtures [5]. In the short term, two trials have demonstrated improved efficacy of dual-active [53] and pyrethroid-PBO treated LLINs [54], suggesting that we are likely to be able to prolong the useful active life of pyrethroid-based interventions. However, the lack of vector control tools with different modes of actions and their increased costs, means that many endemic countries will continue to struggle to develop and implement insecticide resistance management plans. Whilst new products are currently being trialled [55–57], and some have recently come to market, the fine-scale monitoring of resistance phenotypes and mechanisms will be key to mitigating the impacts of insecticide resistance through informed selection of vector control tools.
